# Visual thalamus, “it’s complicated”

**DOI:** 10.1017/S0952523817000311

**Published:** 2017-09-05

**Authors:** DANIEL KERSCHENSTEINER, WILLIAM GUIDO

**Affiliations:** 1 Department of Ophthalmology and Visual Sciences, Washington University School of Medicine, Saint Louis, Missouri 63110; 2 Department of Neuroscience, Washington University School of Medicine, Saint Louis, Missouri 63110; 3 Department of Biomedical Engineering, Washington University School of Medicine, Saint Louis, Missouri 63110; 4 Hope Center for Neurological Disorders, Washington University School of Medicine, Saint Louis, Missouri 63110; 5 Department of Anatomical Sciences and Neurobiology, University of Louisville School of Medicine, Louisville, Kentucky 40292

Often judged as a simple relay, the visual thalamus represents a complex set of subcortical structures that do more than just shuttle retinal signals to visual cortex. Recent evidence suggests that the option offered by Facebook to describe one’s relationship status—“*it’s complicated*”—is a more apt descriptor of visual thalamic structure and function.

In this special issue of *Visual Neuroscience*, we focus on the thalamic structures that receive, process, and transmit information about the visual world. While the dorsal lateral geniculate nucleus (dLGN) is the primary focus of this issue, articles about other thalamic nuclei such as ventral lateral geniculate nucleus (vLGN) and the intergeniculate leaflet (IGL), as well as higher-order nuclei, such as the pulvinar, highlight the multifaceted functions of visual thalamus. Many of the reviews are mouse-centric, underscoring how modern molecular tools allow for unprecedented insights into the cell-type specific circuits that serve vision.

One of the first points of contact between the eye and the brain is the retinogeniculate synapse. **Litvina and Chen** examine this site of communication between a retinal ganglion cell and a thalamocortical relay neuron. They reveal that developing and adult retinogeniculate synapses possess unique structural and biophysical features that work in concert to regulate the gain of information transmission between retina and visual cortex. While the retinogeniculate synapse provides a substrate for the reliable transfer of information, it is also remarkably plastic, with a large dynamic range that can shape the timing and strength of excitatory postsynaptic activity. Such modulation adds a new dimension to retinogeniculate signal transmission, one that contributes to the encoding of complex stimulus features, and regulates tonic and burst firing modes of thalamocortical relay neurons.

All visual information from the retina that reaches the cortex must first pass through the dLGN. **Kerschensteiner and Guido** discuss studies in mouse that show that there is nothing simple about this thalamic relay. Indeed, the advent of transgenic mouse models revealed previously hidden organization of retinal afferents in dLGN, and recent findings indicate that retinal cell-type specific pathways converge onto dLGN neurons in systematic ways to give rise to unconventional receptive field properties that combine binocular information, and encode the direction of stimulus motion, stimulus orientation, irradiance, and other salient features.

**Cox and Beatty** consider the form and function of intrinsic interneurons of dLGN. Few in number and strange in morphology, these neurons receive direct retinal input. Rather than relay signals to visual cortex, they make feed-forward inhibitory connections with thalamorcortical relay neurons. They form one of the most unusual synapses in the brain, making dendro-dendritic connections that bypass the normal route of soma to axon communication, transforming each branch of its dendrite into an independent signal processor to provide both local and global forms of inhibition that contribute to the sharpening of receptive field properties and adjustments in network states.

While retinal inputs provide the primary excitatory drive for dLGN neurons, the overwhelming majority of inputs arise from sources other than the retina. **Hasse and Briggs** discuss the corticogeniculate pathway, a major evolutionarily conserved descending pathway that originates in layer VI of neocortex. This nonretinal projection adjusts retinogeniculate signal transmission to different behavioral states. How such state-dependent gain control is accomplished is an open question. The authors discuss the possibility that corticogeniculate input adjusts stimulus tuning of dLGN neurons to increase the salience of stimuli most relevant in a given behavioral state.

When considering the thousands of retinal axons and countless nonretinal inputs to dLGN, the pattern of synaptic connections onto a dLGN neuron may appear a chaotic tangled web. However, as the review by **Morgan** describes, connectomic approaches show that there is a method to such madness. Indeed, 3-D serial reconstructions of synaptic circuits reveal previously unrecognized higher-order network organization. One intriguing finding from this approach is that mouse dLGN neurons receive more retinal inputs than estimated using electrophysiological measures. This finding not only challenges a widely held belief about retinogeniculate wiring, but also raises important questions about the design of visual pathways and about how retinal signals are combined in the brain to represent the visual world.

In rodents, the dLGN is not the only thalamic target of retinal ganglion cells (RCGs). **Fox and colleagues** discuss the organization and nature of retinal projections to two lesser-known thalamic nuclei, the vLGN and IGL, which reside adjacent to the dLGN, and are part of a “geniculate complex”. Compared to the dLGN, these structures have unique cytoarchitectures and heterogenous cell populations and receive input primarily from RGCs that are intrinsically photosensitive. Unlike dLGN neurons, which project to the visual cortex, vLGN and IGL neurons project to a variety of subcortical structures including the superior colliculus, nuclei of the accessory optic system, and suprachismatic nucleus. Thus, these structures contribute to nonimage forming aspects of vision and are involved in visuomotor control and circadian rhythms.

Perhaps the largest and least understood visual thalamic nucleus is the pulvinar. **Bickford and colleagues** first explore the intricacies of the different subdivisions of the pulvinar and the diversity of inputs they receive. Their review then focuses on the mouse homologue, the lateral posterior nucleus, and discusses regions that receive input from a major afferent source, the superior colliculus. This projection in mice resembles the tectopulvinar projections seen in higher mammals and nonhuman primates. Bickford and colleagues highlight their studies in mice that interrogated projections arising from a specialized cell type known as wide-field vertical cells, which provide powerful excitatory input to the lateral posterior nucleus/pulvinar. This input is widely believed to mediate blindsight, in which subjects with damage to visual cortex can use vision to navigate the world in the absence of conscious visual perception.

Visual information travels from dLGN to cortex along a massive, superhighway, comprised of thousands thalamocortical axons that exit thalamus, course though the internal capsule and form synapses in visual cortex. **Reece and Alonso** discuss how the arrangement of thalamocortical projections from dLGN, along with the evolutionary expansion of the visual cortex, influence visual acuity. Moreover, they propose that as visual cortex increases in size, so does the cortical separation of thalamocortical arbors with overlapping receptive fields, leading to a reorganization of visual cortical maps and the emergence of a pinwheel architecture in the cortical representation of stimulus orientation.

Although these articles may raise as many questions as they answer about visual thalamic structure and function, our hope is they shed some light on the complicated relationship between the outside world and its representation in the brain.

